# Differences in chemosensory response between eyed and eyeless *Astyanax mexicanus* of the Rio Subterráneo cave

**DOI:** 10.1186/2041-9139-4-25

**Published:** 2013-09-04

**Authors:** Jonathan Bibliowicz, Alexandre Alié, Luis Espinasa, Masato Yoshizawa, Maryline Blin, Hélène Hinaux, Laurent Legendre, Stéphane Père, Sylvie Rétaux

**Affiliations:** 1 Equipe Développement Evolution du Cerveau Antérieur, UPR3294 N&D, CNRS, Institut Alfred Fessard, Gif-sur-Yvette F-91198, France; 2 School of Science, Marist College, Poughkeepsie, NY, USA; 3 Department of Biology, University of Maryland, College Park, MD, USA

**Keywords:** Behavior, Cavefish, Evolution, Hybrid population, Micos, Olfaction, Sensory system, Vision

## Abstract

**Background:**

In blind cave-dwelling populations of *Astyanax mexicanus*, several morphological and behavioral shifts occurred during evolution in caves characterized by total and permanent darkness. Previous studies have shown that sensory systems such as the lateral line (mechanosensory) and taste buds (chemosensory) are modified in cavefish. It has long been hypothesized that another chemosensory modality, the olfactory system, might have evolved as well to provide an additional mechanism for food-searching in troglomorphic *Astyanax* populations.

**Findings:**

During a March 2013 cave expedition to the Sierra de El Abra region of San Luís Potosi, Mexico, we tested chemosensory capabilities of the *Astyanax mexicanus* of the Rio Subterráneo cave. This cave hosts a hybrid population presenting a wide range of troglomorphic and epigean mixed phenotypes. During a behavioral test performed *in situ* in the cave, a striking correlation was observed between the absence of eyes and an increased attraction to food extract. In addition, eyeless troglomorphic fish possessed significantly larger naris size than their eyed, nontroglomorphic counterparts.

**Conclusions:**

Our findings suggest that chemosensory capabilities might have evolved in cave-dwelling *Astyanax mexicanus* and that modulation of naris size might at least partially underlie this likely adaptive change.

## Findings

### Introduction

Cave-dwelling animals have long been recognized as excellent models for evolutionary biology [1]. Comparative studies of cave-dwelling (cavefish, CF) and surface-dwelling (surface fish, SF) morphs of the teleost fish *Astyanax mexicanus* have revealed several adaptations to life in dark cave environments [2-5], which have occurred during a few million years of evolution from a common SF-like ancestor [6]. In CF, temporal and spatial modulations in early developmental signaling pathways appear to influence brain development and organization [7-9]. Lateral line neuromasts on the head are more numerous and mediate a special mechanosensory behavior in response to vibrations on the water surface [5]. Chemosensory structures are also modified, as CF possess a higher number of taste buds than their SF counterparts [4,10], and studies in a laboratory setting suggest that laboratory-raised CF originating from the Pachón cave might possess higher olfactory capabilities [11]. Thus, sensory modalities of CF might have evolved as an adaptation to life in dark caves for navigation, mate recognition and food-searching. However, the possibility that these changes might provide a sensory compensation for finding food in the dark has yet to be tested directly in natural cave environments.

### Results

**Figure 1:**
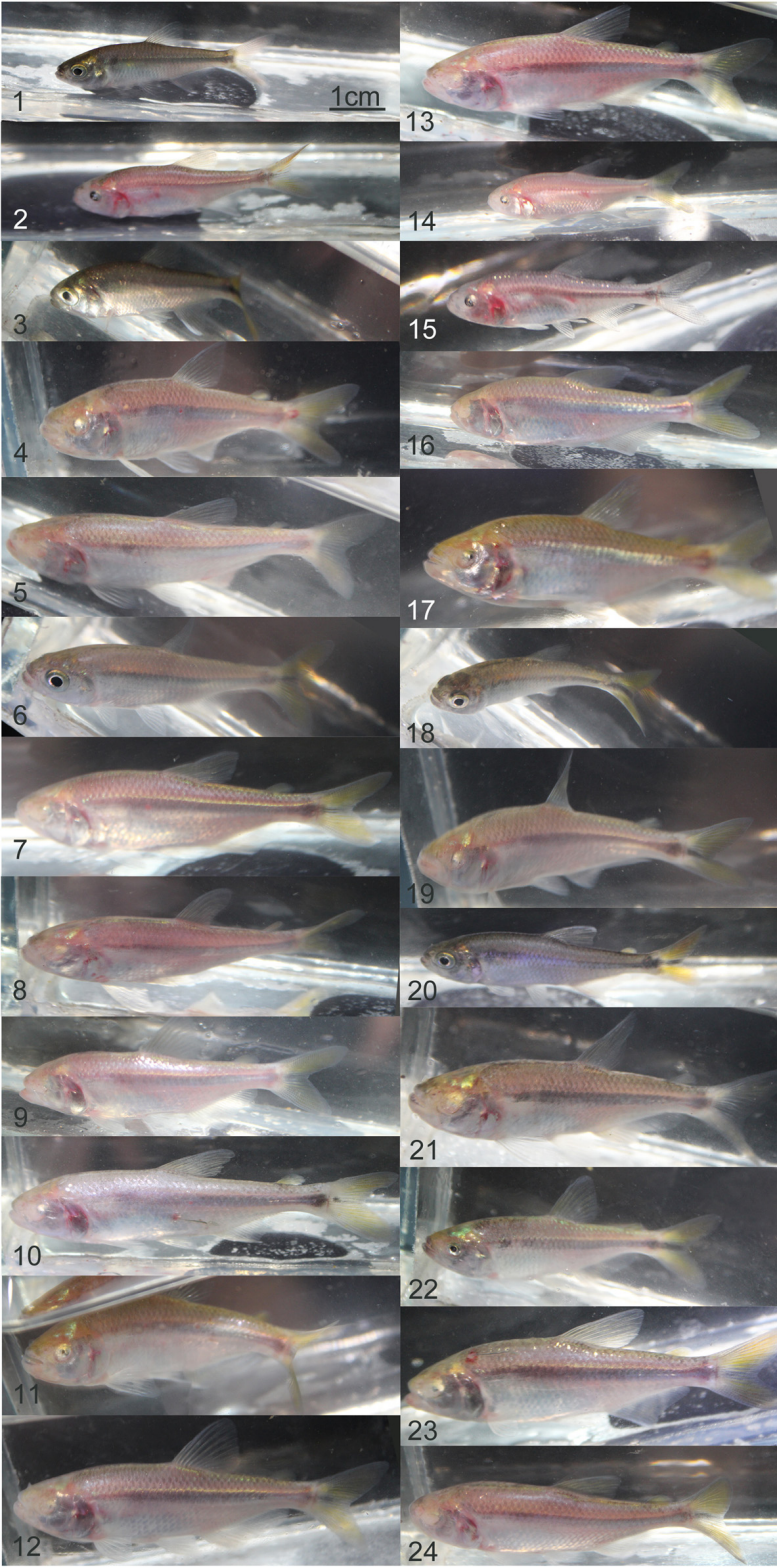
**Subterráneo pools contain a wide array of intermediate, hybrid phenotypes of *Astyanax mexicanus.*** Photographs of our sample of 24 individuals fished with a seine net in the second pool of the Subterráneo cave. Some fish are surface-like (1, 3 and 18; eyed and pigmented), some are totally troglomorphic (5, 7, 8, 9, 10, …; eyeless and depigmented), some display mixed traits (2 and 15 are eyed and depigmented, whereas 21 is eyeless and pigmented) and some show intermediate phenotypes (for example, 17 with low pigmentation and very small eyes). Note that the pigmentation state of fish in a cave is not a good measure of its ability to make pigment cells; therefore, the various degrees of troglomorphy are best described by the size of the eyes, which is highly variable among these 24 individuals (for example, 1, 6, 18 and 20 have big eyes; 2, 3, 15 and 22 have small eyes; 11, 14, 17 and 19 have tiny eyes; and many are eyeless).

The Rio Subterráneo cave is located southwest of the Sierra de El Abra region in the Micos area of Mexico [12,13]. We visited this cave, which is inhabited by bats, in March 2013 (the dry season). We could document that the Subterráneo pools contain a wide array of intermediate hybrid phenotypes that live alongside more troglomorphic fish (Figure 1). This is probably due to seasonal migration of SF into the Subterráneo cave after flooding and subsequent introgression in the existing cave population [12,14,15]. This fact provides a rare possibility to simultaneously test the chemosensory behavior of several morphotypes corresponding to F2 hybrid individuals.

**Figure 2:**
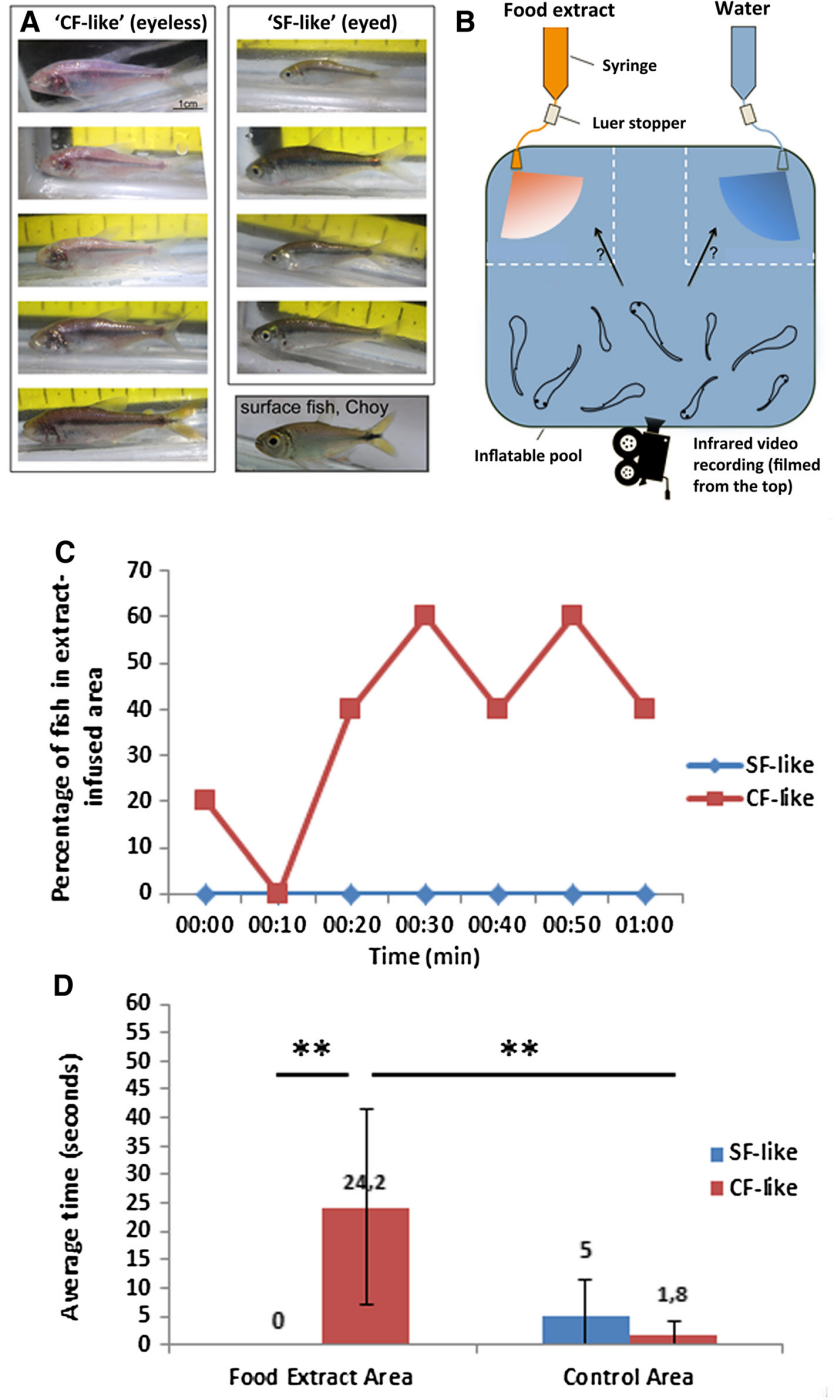
**Behavioral responses to food extract in Subterráneo fish. (A)** Both cavefish (CF)-like troglomorphic and surface fish (SF)-like nontroglomorphic fish were used for the behavioral test. On the bottom right, a SF from the Nacimiento del Río Choy is shown for comparison. **(B)** Schematic of experimental setup. See Methods for details. **(C)** Percentage of SF-like and CF-like fish that were in the food extract–infused quadrant of the pool after solution administration (10-s intervals). **(D)** Average time (in seconds) spent by SF-like and CF-like fish in either extract-infused or control area (**P < 0.01; CF-like = 5, SF-like = 4). Error bars represent standard deviations; indicated numbers correspond to average values.

In this unique context, we set out to test the sensory-dependent food-finding capabilities of Subterráneo fish. To be able to accurately test and quantify this behavior, we chose to collect a representative group of fish and performed our experiment in an enclosed area. The nine fish collected comprised both eyed and eyeless phenotypes, with eyeless fish being also mostly depigmented. The former were classified as SF-like and the latter as CF-like (Figure 2A). Using a specially designed behavioral testing system recently developed in our laboratory and infrared video recording equipment for filming in the dark, we were able to minimize any disturbances to the fish during the experiment. The fish were subjected to a choice test in which food extract was administered to one corner of an enclosed area and water (control) to the other corner (Figure 2B).

**Figure 3:**
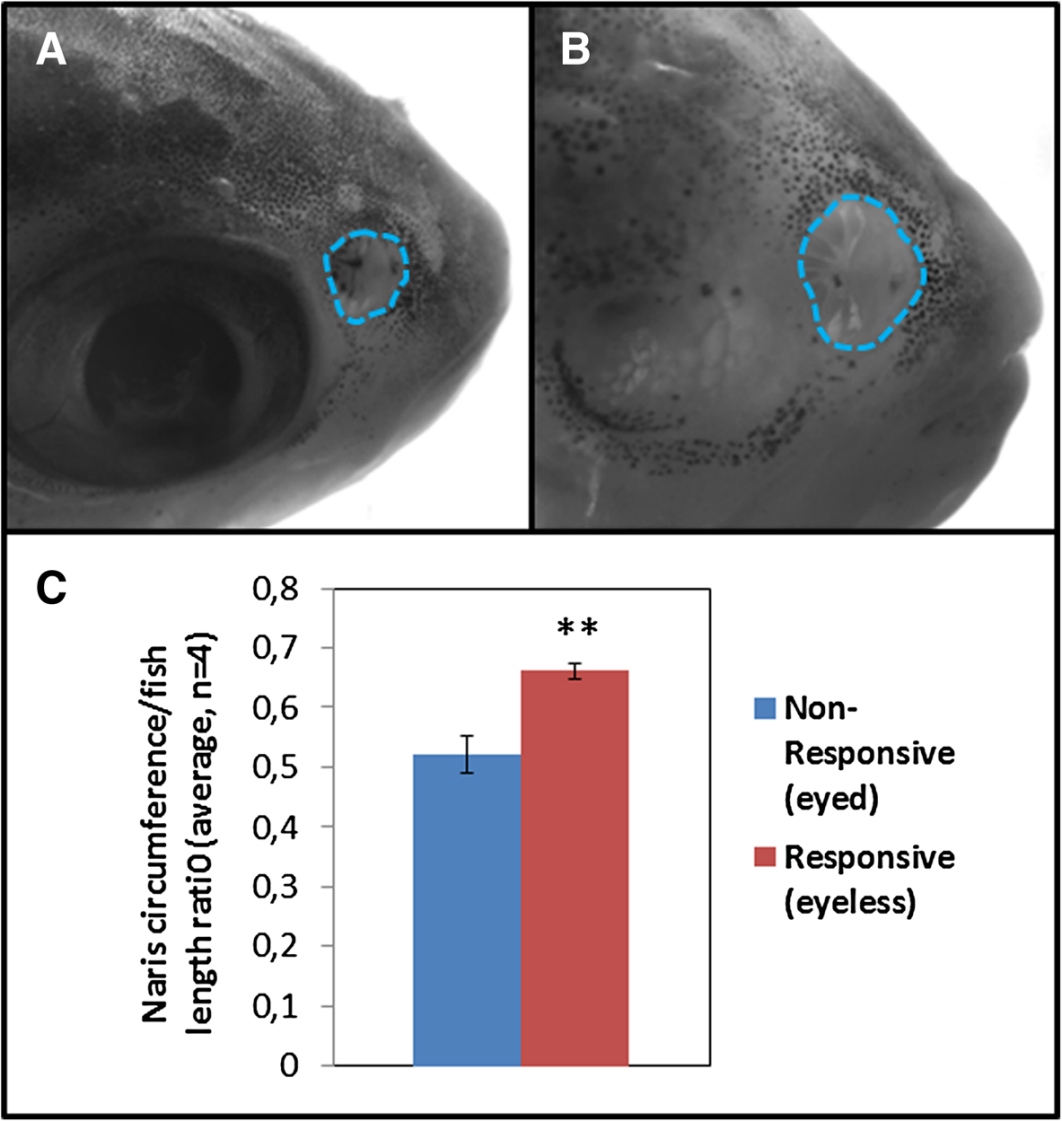
**Naris size is correlated with eye loss and behavioral attraction to food extract.** Measurements of naris circumference (dashed blue encircled areas) on individuals used for behavioral analysis revealed smaller naris size in surface fish-like fish **(A)** than in cavefish -like **(B)** after adjustment to fish body length **(C)** (*n* = 4, *P* < 0.01).

To quantify attraction of fish to the food extract, we analyzed the movie and counted the number of fish that were at the corner of the pool perfused with either the food extract or the water at 10-s intervals during the first minute of the test. Strikingly, the fish that were attracted were all CF-like (Figure 2C). They came to the food source within 20 s after extract administration, continuously swam toward and around the source, occasionally appearing to bite on the tube end (Additional file 1: movie online). They also displayed behavior very similar to the feeding behavior often seen in the laboratory, which includes increased activity combined with a typical feeding body position [16]. Although at any given time a few of the CF-like fish came at the extract-infused corner, each of them approached the odorant tube at some point during the test. They spent an average of 24 s in the extract-infused area, which was significantly more than the 1.8 s spent in the control area during the 1-min test (*P* < 0.01) (Figure 2D). Conversely, none of the SF-like fish approached the food extract corner throughout the test (*P* < 0.01 compared with CF-like fish). Photographs of individual fish taken after the test further revealed significantly larger naris size in CF-like individuals than in SF-like individuals (Figure 3), reflecting an olfactory-related troglomorphic variation in the Subterráneo population.

Our findings reveal an apparent correlation between troglomorphic characteristics and chemosensory-dependent feeding behavior in wild *Astyanax* fish of the Subterráneo cave. Previous studies in a laboratory setting suggested that adult *Astyanax* Pachón cavefish are significantly better than their SF counterparts at finding food in the dark [17]. It has recently been suggested that one of the mechanisms that may contribute to this feeding success in cavefish is their ability to sense vibration using vibration attraction behavior (VAB), a behavior that is mediated through the mechanosensory function of their neuromasts [5]. Since vibrations should be equal on both food extract and control sides of our experimental setup, differences in VAB do not likely contribute to the attraction of CF-like individuals to the food extract that is absent in SF-like fish. Our findings suggest that modified chemosensory capabilities in cavefish do indeed provide a sensory compensation for food-searching in dark cave environments. The fact that heightened chemosensory capabilities has been observed in both laboratory-raised Pachón and wild Subterráneo fish, which come from two independently derived stocks [14], also suggests a possible convergence of this capacity.

Furthermore, the fact that all food extract–responsive fish possessed enlarged nares and strongly reduced eyes shows a correlation between eye loss and modifications in olfactory-related morphology in this population. Although several mechanisms might underlie the observed heightened chemosensory capabilities in CF-like fish, modulations of naris size could be one contributing factor. Incidentally, our results also show that *Astyanax* can respond to a food extract to which they have never been exposed before (in this case, commercial granular fish food). The fact that only the CF-like individuals responded to this novel food might reflect a more generalist food detection ability of troglomorphic fish. If confirmed, such opportunism for multiple food sources might reflect a troglomorphic adaptation to an environment where seasonal water flows create sudden changes in nutrient provision.

### Conclusions

To the best of our knowledge, this report is the first report to describe a behavioral experiment performed on wild *Astyanax* in their natural environment. The results obtained raise the intriguing possibility that olfactory capabilities might have evolved in cave-dwelling *Astyanax*, and it is tempting to propose that this change improves food-finding in dark environments, where vision is useless. Additional studies are certainly necessary to better characterize the observed differences in chemosensory response and to test whether they correspond to bona fide sensory adaptations in *Astyanax mexicanus* (and potentially other cave-dwelling animals as well).

### Methods

#### Sampling and photography

For assessment of the phenotypes encountered in the Subterráneo cave, fish were caught with a seine net and transferred to inflatable plastic pools. They were individually photographed with a Canon EOS 650D camera (Canon U.S.A., Melville, NY, USA) in a narrow glass box and returned to the pool. Fieldwork was performed under the auspices of Mexican permit 02241/13 (to SR) delivered by Secretaria de Medio Ambiente y Recursos Naturales.

#### Behavioral test

To collect fish for behavioral testing, a meat-baited minnow trap was set up overnight. The collected fish were placed in a 61-cm × 61-cm × 15-cm inflatable pool and were left for an acclimatization period of 48 h. Two 50-ml syringes were attached to a stationary tripod and connected to medical solution administration tubing containing a Luer stopper to control solution flow (Baxter, Thetford, UK). The ends of the two administration sets were attached to opposite corners of the plastic pool (Figure 2B). Food extract was prepared by adding 5 g of granular fish food (TetraDiskus; Tetra, Blacksburg, VA, USA) to 50 ml of local water, mixing, and filtering using glass microfiber filters (Whatman plc, Kent, UK) to remove any food particles. This solution was added to one syringe while a water control was added to the other. Solution flow from the food extract and control samples was initiated simultaneously, and the experiment was filmed from the top using a Sony DCR*-*SR200 Handycam camcorder equipped with NIGHTSHOT mode (Sony Electronics, San Diego, CA, USA). Food extract and control areas were defined arbitrarily as 20-cm × 20-cm zones adjacent to the two respective tube ends. To measure the time each fish spent in the food extract area, individual fish were manually tracked through frame-by-frame analysis of the movie using VirtualDub and ImageJ software (National Institutes of Health, Bethesda, MD, USA). A Mann–Whitney *U* test was utilized to determine the statistical significance of the results. After the test, the fish were collected, photographed individually and returned to the pool.

#### Naris size measurements

Naris circumference and standard body length [18] were measured using ImageJ software and reported as a ratio to control for size variation between individuals.

## Competing interests

The authors declare that they have no competing interests.

## Authors’ contributions

JB, AA, LE, MY, MB, HH, LL, SP and SR prepared for and performed field experimentation. AA performed the test and recorded the movie, alone, in the dark. JB and AA analyzed the data. JB, AA and SR wrote the paper. All authors read and approved the final manuscript.

## Supplementary Material

Additional file 1 **Recording behavior in the Rio Subterráneo cave.** A one minute movie (infrared recording) shows attraction of “CF-like” but not “SF-like” fish to food-related odors. The “food” tubing is at the top left, and the “control water” tubing is at the top right of the plastic pool. Click here for file
